# Relationship Between Screen Time and Dry Eye Symptoms During the COVID-19 Pandemic in the Pediatric Population of the Western Region of Saudi Arabia

**DOI:** 10.7759/cureus.31015

**Published:** 2022-11-02

**Authors:** Wejdan Alnahdi, Manal Hadrawi, Enam Danish, Amer Alghamdi, Nada Taher, Albaraa T Alfaraidi, Nourah Alageel

**Affiliations:** 1 Department of Ophthalmology, King Abdulaziz Medical City, Ministry of the National Guard - Health Affairs, Jeddah, SAU; 2 Pediatric Ophthalmology and Strabismology, Department of Ophthalmology, King Fahad Armed Forces Hospital, Jeddah, SAU; 3 Department of Ophthalmology, College of Medicine, King Saud University, Riyadh, SAU; 4 Department of Ophthalmology, College of Medicine, King Saud Bin Abdulaziz University for Health Sciences, Jeddah, SAU; 5 Department of Family Medicine, King Abdulaziz Medical City, Ministry of the National Guard - Health Affairs, Jeddah, SAU

**Keywords:** covid 19, osdi questionnaire, screen time, pediatric dry eye, dry eye symptoms

## Abstract

Objective

To measure the prevalence of dry eye disease (DED) and study the relationship between screen time and dry eye symptoms in the pediatric population during the coronavirus disease 2019 (COVID-19) pandemic using the Ocular Surface Disease Index (OSDI) questionnaire.

Methods

In this descriptive, observational, cross-sectional study, our survey included the pediatric population, ages 1 to 18 years, of both genders, who attended outpatient clinics of two main hospitals in Jeddah, Saudi Arabia. Collected data included age, gender, dry eye symptoms, and common DED risk factors, followed by the Ocular Surface Disease Index (OSDI) questionnaire, which consists of 12 items graded on a five-point scale (0 = never to 4 = all the time).

Results

A total of 329 pediatric participants were included, with more than half of the participants (56.1%) males and 58.5% aged 12-18 years. The most frequently reported symptoms (reported as often or always) were decreased vision (23.0%) and itchy eyes (22.1%). Environmental factors have an effect on developing DED symptoms, as some participants (21.8%) have reported being uncomfortable in windy weather and 15.8% have reported this in places with air conditioners. Based on the OSDI diagnostic criteria, 250 (76.1%) participants had DED. Furthermore, in terms of severity, 44 (13.3%) participants had mild DED, 62 (18.8%) participants had moderate DED, and 145 (43.9%) participants had severe DED. We found that prolonged exposure to mobile screens for two to three hours or four hours or more was associated with a higher DED incidence compared to those exposed for shorter periods. Older age categories were more likely to experience DED (80.8% and 78.2% in age categories 12-18 and 7-12, respectively, versus 57.6% in the youngest age category (p = 0.001)). Additionally, DED was independently associated with participants with a previous history of eyeglasses prescription and those experiencing dry eyes while using electronic devices.

Conclusion

Since many children use electronic devices for education and entertainment, we found that symptoms of DED due to prolonged screen time have increased among the pediatric population during the COVID-19 pandemic. Therefore, awareness efforts should be directed to reduce the rate of controllable risk factors like personal computer use. In addition, educational campaigns are warranted to provide all possible preventive measures against DED, especially to children with uncontrollable risk factors for developing DED.

## Introduction

Dry eye disease (DED), also known as keratoconjunctivitis sicca (KCS) and keratitis sicca, is a multifactorial, painful inflammatory disorder of the tear film that occurs due to insufficient lubrication by the eyes’ tears [1]. It is attributed to the loss of homeostasis of the tear film, which is found on the outermost corneal surface, leading to various ocular symptoms due to increased evaporation or decreased secretion of the tear film. The disturbance of the tear film homeostasis is thought to be due to eyelid abnormalities, deficits in tear constituents, or inadequate blinking secondary to prolonged time screen [2]. This could also be due to systemic illnesses, allergies, eye surgeries, or autoimmune disorders. Regardless of the cause, DED causes unpleasant symptoms that range from a burning sensation to eye discomfort, pain, photophobia, squint, accommodation spasms, or blurred vision, which eventually affect the quality of life. In addition, DED has been associated with depression, stress, and anxiety, making it a debilitating eye disease [2]. Severe DED increases the risk of developing conjunctivitis, headache, eye infection, corneal abrasion, and ulcers [1-4]. The diagnosis of DED is usually based on subjective symptoms and clinical dry eye examination findings. Signs and symptoms of DED may mimic those of other ocular surface diseases such as infection, allergic conjunctivitis, keratopathy, and episcleritis. The diagnosis of DED is challenging in clinical practice, which leads to a delay in diagnosis until an advanced stage of the disease has ensued.

Additionally, the COVID-19 pandemic has considerably changed people’s lifestyles, especially those of children. That is, governments have been forced to adopt safety precautions to combat the transmission of this novel virus. Due to such preventive measures, schools and academic activities worldwide switched to a virtual learning modality and paperless classrooms. Since this pandemic, more young people have had dry eye and other eye symptoms, mainly due to in-house quarantine, limited outdoor activities, and increased screen time [5]. In other words, prolonged and steady use of visual display terminals (VDTs) or digital screens for professional and recreational purposes has been increasing across all age groups. The use of digital screens is a significant and well-known precipitating factor for DED. This is attributed to the extended use of VDTs with short watching distances, which causes less frequent and incomplete blinking. This, hence, promotes tear film instability by accelerating tear film evaporation [2].

Although this relationship in adults has been well-studied [6], few studies have reported the prevalence of dry eye disease among children during the COVID-19 pandemic using different assessment tools [6-7]. A cross-sectional study performed at pediatric ophthalmology clinics at two institutes in Egypt studied the effect of the COVID-19 pandemic on screen time and its relationship with DED symptoms in a pediatric population using the Standard Patient Evaluation of Eye Dryness (SPEED) questionnaire [6]. They concluded that the increased screen time in children during the COVID-19 pandemic might contribute to the symptoms of DED. Therefore, this study aims to measure the prevalence of dry eye disease in the pediatric population during the COVID-19 pandemic and other attributing factors using the Ocular Surface Disease Index (OSDI) questionnaire.

## Materials and methods

A descriptive observational cross-sectional study was conducted using a self-administrated questionnaire targeting all pediatric populations of both genders, aged 10-18 years, attending outpatient pediatric ophthalmology clinics at King Fahd Armed Forces Hospital and King Abdulaziz Medical City, Jeddah. The data were collected from October 2021 to January 2022. The estimated sample size was calculated using Roasoft, Inc (Seattle, WA). The required sample size of 329 children was deemed sufficient to provide a one-sided 95% confidence level, with an estimated 50% response distribution and an error margin of ±5%.

A questionnaire translated into Arabic was given to the children or their parents. Informed consent was obtained from the legal guardian of each child before the survey, but after elucidating the confidentiality measures and the study's objectives. No inducements were provided. Ethical approval was obtained from the ethics review committee of the Armed Forces Scientific Research Center, Saudi Arabia. Confidentiality was maintained thoroughly, as no personal information was obtained and all questionnaires were kept anonymous. A nonprobability convenience sampling technique was used, as any child aged 1 to 18 years who attended the pediatric outpatient clinics was included in the study.

OSDI questionnaire and score calculation

The OSDI is a questionnaire specific to DED that measures the severity of DED-related symptoms. The questionnaire consists of 12 items graded on a five-point scale (0 = Never to 4 = all the time). The questionnaire was divided into three sections: vision-related functions, ocular symptomatology, and environmental triggers [8]. Scores of each section were computed, and a final raw score was obtained by summing up the section scores. The raw final score ranged between 0 and 48. A final percentage score was calculated using the formula: (raw score*25)/the number of questions answered [9]. Participants with normal eyes scored an OSDI score of 0-12 while those with mild, moderate, and severe DED scored 13-22, 23- 32, and > 33, respectively.

Statistical analysis

The Statistical Package for the Social Sciences (IBM Corp. Released 2019. IBM SPSS Statistics for Windows, Version 26.0. Armonk, NY: IBM Corp) was used for statistical analysis. The reliability of the overall OSDI questionnaire items and the OSDI subscales was assessed statistically using Cronbach's alpha test. Descriptive statistics were adopted to express categorical (frequencies and percentages) and numerical data (medians and interquartile ranges [IQRs]). The relationship between DED and demographic, clinical, and lifestyle characteristics was investigated using a chi-squared test. The significant variables from the univariate analysis were subsequently used in a logistic regression model, using the enter method, to assess the risk factors for DED. The dependent variable was DED status (no versus yes), and the significantly associated variables were used as independent variables. Results were expressed as odds ratios (ORs) and their respective 95% confidence intervals (95% CIs). A p-value of < 0.05 was used to indicate statistical significance.

## Results

Participants’ demographics, characteristics, and clinical history

A total of 336 responses were initially received; however, seven were omitted due to missing data. Therefore, 329 pediatric patients were included in the current study. More than half of the participants were males (56.1%) and aged 12-18 (58.5%). A history of chronic diseases was found in 8.8% of all participants, and 7.9% have used medications daily. In addition, approximately one-quarter (23.0%) of the respondents had allergic conditions (asthma, allergic rhinitis, allergic eczema, food allergy, or allergic conjunctivitis), and 15.2% received anti-allergic medications. Regarding the ophthalmologic history, 8.8% of the participants had received a medical/surgical intervention for eye disease, and 3.9% had undergone eye surgery in the past six months. More details about the clinical history of patients are listed in Table 1.

**Table 1:** Characteristics and clinical history of participants (n=329)

Parameter	Category	Frequency	Percent
Gender	Male	185	56.1
	Female	144	43.6
Age	1-6 y	59	17.9
	7-12 y	78	23.6
	12-18 y	192	58.5
Chronic diseases	Yes	29	8.8
Use of daily medications	Yes	26	7.9
Medical/surgical eye treatment	Yes	29	8.8
Eye surgery during the last 6 months	Yes	13	3.9
Current conjunctivitis	Yes	20	6.1
Diagnosed with any eye disease	Yes	55	16.7
Prescribed eyeglasses?	Yes	102	30.9
Dry eye while using electronic	Yes	75	22.8
Allergies	Yes	76	23.0
Use of any anti-allergic medication	Yes	50	15.2

Dry eye symptoms and vision-related function

**Figure 1:**
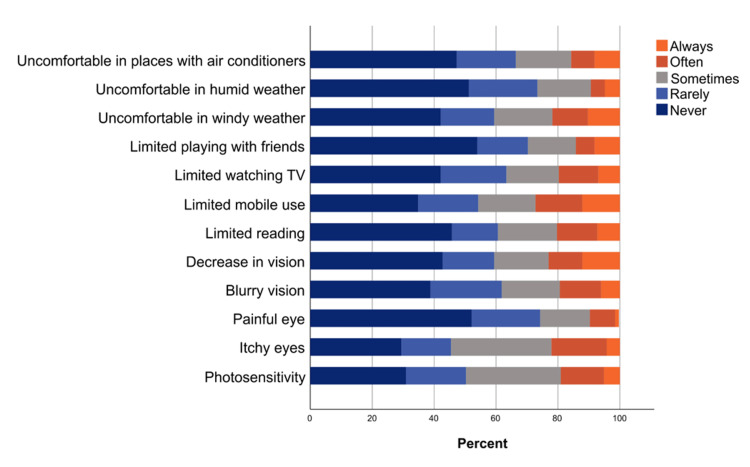
The percentages of participants' responses to different items of the OSDI questionnaire OSDI: Ocular Surface Disease Index

**Figure 2:**
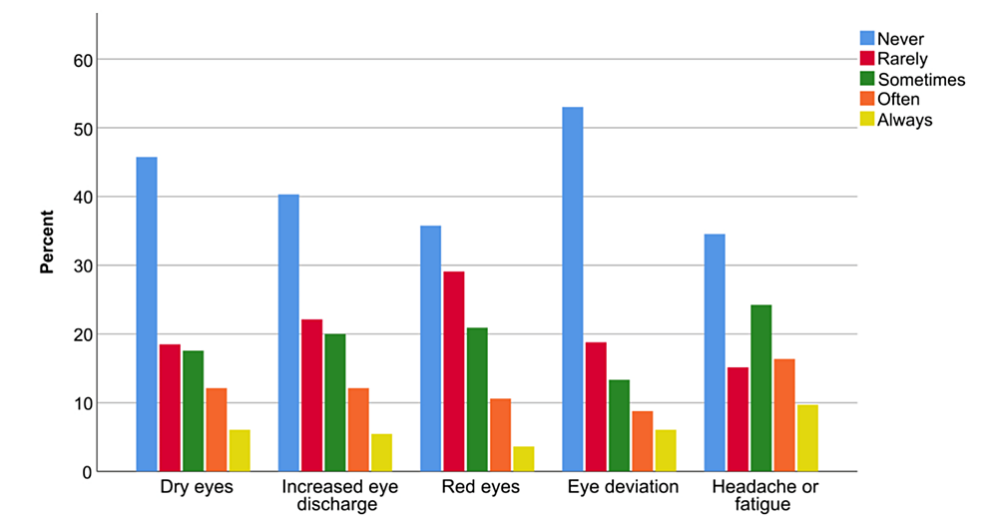
The percentages of participants’ responses to selected eye symptoms

The detailed responses of participants to the different items of the OSDI questionnaire are depicted in Figure 1. The most frequently reported symptoms (reported as often or always) were decreased vision (23.0%) and itchy eyes (22.1%). Moreover, dry eye symptoms have been reported to cause discomfort to some participants during mobile use (27.3%) and reading (20.3%). Regarding environmental factors, participants felt uncomfortable in windy weather (21.8%) and places with air conditioners (15.8%). There were other reported symptoms, although they are not included in the OSDI. The most-reported ones (reported as often or always) were headache/fatigue (26.1%), dry eyes (18.2%), and increased eye discharge (17.6%, Figure 2).

Results of the OSDI scale and subscales

**Figure 3:**
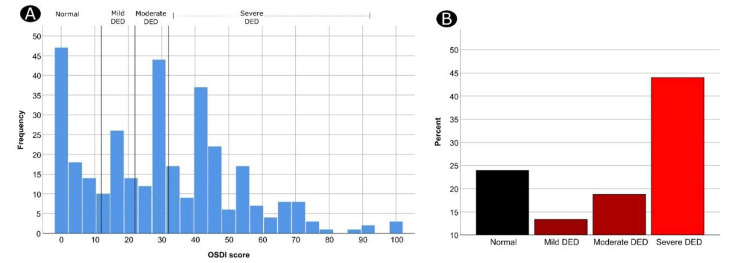
Frequency distribution of OSDI scores among the participants (A) and the different OSDI categories of DED (B) OSDI: Ocular Surface Disease Index; DED: dry eye disease

In general, the three OSDI subscales showed robust to reliable statistical reliability (with a Cronbach’s alpha between 0.805 and 0.862) [10]. Additionally, all OSDI questionnaire items were reliable (Cronbach’s alpha = 0.907). The median (IQR) overall OSDI score for all the participants was 29.2 (12.5-44.3) (Table 2). Based on the OSDI diagnostic criteria, 79 (23.9%) participants had no DED, whereas 250 (76.1%) patients had DED. Furthermore, 44 (13.3%) participants had mild DED, 62 (18.8%) had moderate DED, and 145 (43.9%) had severe DED (Figure 3).

**Table 2:** Descriptive statistics of the OSDI scale and subscales OSDI: Ocular Surface Disease Index

Parameter	Number of Items	Minimum - Maximum	Cronbach's Alpha	Median (IQR)
Eye symptoms	5	0-20	0.805	6.0 (2.8-9.0)
Vision-related function	4	0-16	0.862	4.0 (0.0-8.0)
Environmental triggers	3	0-12	0.814	3.0 (0.0-6.0)
Raw OSDI	12	0-48	0.907	14.0 (6.0-21.3)
Percent OSDI	12	0-100	0.907	29.2 (12.5-44.3)

Factors associated with DED

Results of the univariate analysis showed that increased age was associated with DED (80.8% and 78.2% in age categories 12-18 and 7-12, respectively, versus 57.6% in the youngest age category (*p* = 0.001). Furthermore, compared to their counterparts, DED was significantly higher among participants with a positive history of using daily medications (92.3% versus 74.6%, *p *= 0.042), medical/surgical eye treatment (96.6% versus 74.1%, *p *= 0.007), conjunctivitis (95% versus 74.8%, *p *= 0.041), an eye disease (92.7% versus 72.7%, *p *= 0.002), allergy (90.8% versus 71.7%, *p* = 0.001), and dry eye while using electronic devices (97.3% versus 69.7%, *p *< 0.0001). Moreover, DED was found significantly high in participants who use eyeglasses (94.1% versus 68%, *p* < 0.0001), those who cannot close their eyelids (100% versus 75.1%,* p* = 0.039), and those currently using anti-allergic medications (90% versus 73.6%, *p* = 0.012) or eye drops for conjunctivitis (100% versus 72.6%, *p *< 0.0001, Table 3).

**Table 3:** Demographic and clinical factors associated with dry eye disease according to OSDI score DED: dry eye disease; OSDI: Ocular Surface Disease Index; **means *P* < .01; * means .01 < *P* < .05

Variable	Category	Normal (0-12 points) N (%)	Symptomatic DED (≥13 points) N (%)	P
Age (yrs)	1-6 y	25 (42.4)	34 (57.6)	0.001**
	7-12 y	17 (21.8)	61 (78.2)	
	12-18 y	37 (19.2)	156 (80.8)	
Gender	Male	50 (27.0)	135 (73.0)	0.147
	Female	29 (20.1)	115 (79.9)	
Chronic diseases	No	75 (24.9)	226 (75.1)	0.180
	Yes	4 (13.8)	25 (86.2)	
Use of daily medications	No	77 (25.4)	226 (74.6)	0.042*
	Yes	2 (7.7)	24 (92.3)	
Medical/surgical eye treatment	No	78 (25.9)	223 (74.1)	0.007**
	Yes	1 (3.4)	28 (96.6)	
Eye surgery during the last 6 months	No	78 (24.6)	239 (75.4)	0.161
	Yes	1 (7.7)	12 (92.3)	
Conjunctivitis	No	78 (25.2)	232 (74.8)	0.041*
	Yes	1 (5.0)	19 (95.0)	
Diagnosed with any eye diseases	No	75 (27.3)	200 (72.7)	0.002**
	Yes	4 (7.3)	51 (92.7)	
Prescribed eyeglasses	No	73 (32.0)	155 (68.0)	< 0.0001**
	Yes	6 (5.9)	96 (94.1)	
Cannot close the whole lid	No	79 (24.9)	238 (75.1)	0.039*
	Yes	0 (0.0)	13 (100.0)	
Dry eye while using electronic devices	No	77 (30.3)	177 (69.7)	< 0.0001**
	Yes	2 (2.7)	73 (97.3)	
Presence of Allergic conditions	No	72 (28.3)	182 (71.7)	0.001**
	Yes	7 (9.2)	69 (90.8)	
Use of any anti-allergic medication	No	74 (26.4)	206 (73.6)	0.012*
	Yes	5 (10.0)	45 (90.0)	
Used drops for conjunctivitis	No	79 (27.4)	209 (72.6)	< 0.0001**
	Yes	0 (0.0)	41 (100.0)	

Regarding the effect of lifestyle factors and screen time on DED, we found that prolonged exposure to mobile screens for two to three hours in 78.1% or four hours or more in 81.8% was associated with more DED incidence compared to those who were exposed for shorter periods (62.3% and 72.9% for those exposed for one to two hours and zero to one hour, respectively, *p* = 0.024). Although there was also a significant difference (*p* = 0.007, Table 4) in exposure to TV screens, with higher proportions of participants with DED who watched TV for one to two hours (84.8%) compared to those who watched TV for zero to one hour (77.7%), two to three hours (60.3%), or four or more hours (77.7%). We cannot conclude that watching TV is a risk factor for DED, as the minimum watching TV hours are almost equal to the maximum watching hours in terms of DED incidence.

**Table 4:** Lifestyle-related factors associated with DED according to OSDI score DED: dry eye disease; OSDI: Ocular Surface Disease Index; **means *P* < .01; * means .01 < *P* < .05

Variable	Category	Normal (0-12 points) N (%)	Symptomatic DED (≥13 points) N (%)	p
Time spent using the mobile	0-1h	13 (27.1)	35 (72.9)	0.024*
	1-2h	23 (37.7)	38 (62.3)	
	2-3h	16 (21.9)	57 (78.1)	
	4h and more	27 (18.2)	121 (81.8)	
Time spent using electronic devices	0-1h	45 (29.8)	106 (70.2)	0.137
	1-2h	13 (21.3)	48 (78.7)	
	2-3h	10 (18.2)	45 (81.8)	
	4h and more	11 (17.5)	52 (82.5)	
Time spent watching TV	0-1h	31 (22.3)	108 (77.7)	0.007**
	1-2h	12 (15.2)	67 (84.8)	
	2-3h	25 (39.7)	38 (60.3)	
	4h and more	11 (22.4)	38 (77.6)	
Time spent on reading	0-1h	52 (26.9)	141 (73.1)	0.260
	1-2h	12 (15.8)	64 (84.2)	
	2-3h	7 (21.9)	25 (78.1)	
	4h and more	8 (27.6)	21 (72.4)	
Time doing outdoor activities	0-1h	27 (22.5)	93 (77.5)	0.336
	1-2h	16 (20.8)	61 (79.2)	
	2-3h	19 (22.9)	64 (77.1)	
	4h and more	17 (34.0)	33 (66.0)	
Sleep time	1-5h	8 (17.0)	39 (83.0)	0.331
	6-8h	45 (26.9)	122 (73.1)	
	8h and more	26 (22.4)	90 (77.6)	

Risk factors for DED

The outcome of the regression analysis showed that the participants in the older age categories were more likely to experience DED (OR = 2.79, 95%CI, 1.09 to 7.12, *p *= 0.032 for the 7-12 age category and OR = 3.20, 95%CI, 1.37 to 7.48, *p* = 0.007 for the 12-18 age category). Additionally, DED was independently associated with participants with a previous history of eyeglasses prescription (OR = 4.41, 95%CI, 1.32 to 14.72,* p* = 0.016) and those experiencing dry eye while using electronic devices (OR = 6.74, 95%CI, 1.43 to 31.70, *p* = 0.016). However, participants who watched TV for two to three hours were less likely to experience DED symptoms (OR = 0.45, 95%CI, 0.21 to 0.97, *p *= 0.043, Table 5).

**Table 5:** Risk factors for dry eye disease among the participants OR: odds ratio; CI: confidence interval. **means *P* < .01; * means .01 < *P* < .05

Parameter	Category	OR (95%CI)	P
Age	1-6 y	Reference	
	7-12 y	2.79 (1.09-7.12)	0.032*
	12-18 y	3.20 (1.37-7.48)	0.007**
Use of daily medications	No	Reference	0.990
	Yes	1.01 (0.14-7.53)	
Medical/surgical eye treatment	No	Reference	0.361
	Yes	2.95 (0.29-29.90)	
Conjunctivitis	No	Reference	0.734
	Yes	1.49 (0.15-15.08)	
Diagnosed with any eye diseases	No	Reference	0.653
	Yes	1.44 (0.30-6.94)	
Prescribed eyeglasses	No	Reference	0.016*
	Yes	4.41 (1.32-14.72)	
Dry eye while using electronic	No	Reference	0.016*
	Yes	6.74 (1.43-31.70)	
Presence of allergies	No	Reference	0.109
	Yes	3.65 (0.75-17.76)	
Use of any anti-allergic medication	No	Reference	0.481
	Yes	0.50 (0.07-3.42)	
Time spent using the mobile	0-1h	Reference	
	1-2h	0.50 (0.18-1.39)	0.187
	2-3h	1.02 (0.36-2.85)	0.975
	4h and more	0.82 (0.30-2.21)	0.691
Time spent watching TV	0-1h	Reference	
	1-2h	1.99 (0.88-4.48)	0.096
	2-3h	0.45 (0.21-0.97)	0.043*
	4h and more	0.54 (0.20-1.43)	0.214

## Discussion

The prevalence of DED in children has been increasing in the last few years with the popularity of electronic device use. The global prevalence of DED, according to the Dry Eye Workshop (DEWS) II in 2017, ranged from 20% to 50% [11]. DED causes variable symptoms that may not correlate with the severity of the disruption in the ocular surface. The disease is progressive and may lead to permanent ocular surface damage if left untreated [12]. According to the Tear Film Ocular Surface Society (TFOS) Dry Eye Workshop (DEWS) II criteria, DED has been categorized as either aqueous deficient or evaporative dry [13]. Clinically, patients often present with symptoms of both types of the disease.

During the COVID-19 lockdown in several countries, all academic activities of schools and universities were made online, which led to prolonged eye exposure to screens and electronic devices [14]. The latest recommendations from the AAP advise caregivers to limit screen time in children aged two to five years old to one hour or less each day [15]. However, the Royal College of Paediatrics and Child Health (RCPCH) in their recent 2019 guidance recommended that the amount of time spent on devices should be tailored to each child [16]. The OSDI is a clinical instrument that allows physicians to measure DED-related symptoms' severity and understand their impact on visual function and daily life with a sensitivity of 60% and a specificity of 79%. In comparison to the short form 12-Health Survey (SF-12) and McMonnies Questionnaire (MQ), the OSDI has a higher sensitivity and specificity [9]. The current study aimed to measure the prevalence of dry eye disease and its impact on visual function in the pediatric population during the COVID-19 pandemic using a web and hardcopy-based OSDI questionnaire.

In our study, 329 children participated in filling out the OSDI form. Based on the OSDI diagnostic criteria, 250 (76.1%) participants had symptomatic DED (OSDI score above 22), whereas 79 (23.9%) participants had no DED. We also found that 145 (43.9%) children reported severe DED symptoms. These findings are comparable to the D. García-Ayuso et al. study who assessed the prevalence of dry eye symptoms among university students during COVID-19 using OSDI. In their research, the prevalence of symptomatic DED (OSDI score above 22) among 676 participants was 51.8% [7].

Furthermore, Neti et al., who evaluated the impact of COVID‐19 health measures on dry eye symptoms, found that young to middle-aged participants reported worsening dry eye symptoms during the lockdown. They proposed that the lifestyles of the elderly were not significantly altered as much as younger individuals [16]. Their study found two independent risk factors for DED increased VDT usage and female gender. These findings were consistent with another study by D. García-Ayuso et al., which found that the prevalence of DED was higher in females [7]. In contrast, our current study found no statistically significant difference between both genders. However, we found that children who used smartphones for four hours or more had a statistically significant increase in DED incidence. In addition, we found that children with a previous history of eyeglasses prescription had a higher incidence of dry eye symptoms. In previous studies, increased screen time has been associated with a higher incidence of dry eye symptoms in the pediatric population [4]. The Osaka study by Uchino and colleagues demonstrated that patients with definite DED who had prolonged exposure to VDT had lower mucin secreted by goblet cells in the conjunctiva [17].

Strengths and limitations

This is the first study to assess the prevalence of dry eye disease among the pediatric Saudi population in the COVID-19 era. In addition, our findings may provide helpful information to the health authorities regarding the impact of implementing restrictions on educational institutions on children and adolescents. Also, the large sample size allows our findings to be generalized to other populations. One of the limitations of this study is that the influence of environmental factors on the development of dry eye symptoms was not considered. Furthermore, the reported symptoms of DED in the survey may be caused by other ocular surface diseases. Self-reporting, as in any questionnaire, can have recall bias. When concluding the findings, it is essential to consider that some children often have difficulty expressing dry eye symptoms.

## Conclusions

This study highlights the importance of recognizing DED in its early stages, as severe forms can become chronic and more challenging to treat. Since many children use electronic devices for education and entertainment, we found that symptoms of DED due to prolonged screen time have increased among the pediatric population during the COVID-19 pandemic. Therefore, awareness efforts should be directed to reduce the rate of controllable risk factors like personal computer or digital device use. In addition, educational campaigns are warranted to provide all possible preventive measures against DED, especially to children with uncontrollable risk factors for developing DED.
